# Supplementary material for: Are carbon emissions trading and green financial instruments synergistic? -Comprehensive quantitative research based on content analysis
Source: PLoS One. 2024 Mar 7;19(3):e0298601. doi: 10.1371/journal.pone.0298601 (PMC10919601; doi:10.1371/journal.pone.0298601)
Supplement: S1 Data — (ZIP) [file pone.0298601.s001.zip › Data/地级市Z_F工作报告.zip!/║■─╧/│ú╡┬╩╨/└┤╫╘╣1⁄2╓┌║┼í╛┬φ┐╦╩2╛▌═°í┐_2019.docx]

各位代表：

现在，我代表市人民政府，向大会报告工作，请予审议，并请各位政协委员和其他列席人员提出意见。

一、2018年工作回顾

2018年，我们坚持以习近平新时代中国特色社会主义思想为指引，在省委省政府和市委的坚强领导下，在市人大、市政协的监督支持下，大力推进开放强市产业立市战略，坚定不移打好三大攻坚战，较好地完成了市七届人大二次会议确定的目标任务，保持了经济社会平稳健康发展。地区生产总值超过3400亿元（预计数，下同），增长8%以上；一般公共预算收入超过261亿元，增长6.5%以上；固定资产投资增长10%以上。

一年来，我们大力兴产业、强实体，发展质量有效提升。全面推进产业立市三年行动，着力振兴实体经济，高质量发展态势正在形成。产业投资占固定资产投资达51%，地方财政收入中税收占比达69.5%。

产业升级步伐加快。传统产业变速换挡，新兴产业快速成长。规模以上工业增加值增长7%以上，新增规模以上工业企业159家，工业投资、工业技改投资、高新技术产业投资分别增长25%、28%、16%，高新技术产业产值突破1200亿元，新增高新技术企业67家，21家企业被评为湖南省小巨人企业，飞沃新能源获批国家智能制造试点示范项目。农村一二三产业加速融合发展，成功创建国家农业科技园区，新增家庭农场1097个、农民合作社973家、规模以上农产品加工企业31家、“两品一标”认证117个，休闲农业经营收入增长15%。服务业对经济的拉动作用持续增强，新增规模以上服务业企业121家，社会消费品零售总额达1260亿元、增长10.6%。

产业载体支撑强化。优化园区产业布局，明确国家级园区不超过“三主三特”、省级园区不超过“两主两特”的产业定位，启动一批特色园区建设。改善园区基础条件，完成基础建设投入102亿元，建设标准化厂房220万平方米。加快释放园区活力，向常德经开区、常德高新区下放管理权限199项，所有区县（市）园区全面启用“2号公章”，全市11个园区与发达地区17个园区（商会）成功结对合作，常德经开区获批“湖南省军民融合产业示范基地”，澧县经开区获批省级高新区。全力主攻产业项目，35个项目列入全省“五个100”项目范围，新开工亿元以上产业项目214个，其中10亿元以上项目15个、工业项目128个，粤港模科（一期）、金富力锂电池、溢多利生物、浩天翼无人机等项目建成投产，烟厂易地技改、中联重科起重机械产业基地、三一常德产业园等项目扎实推进。

产业品牌影响扩大。深入推进“常德品牌·中国品质”行动，实现以品牌品质提升促产业质量提升、城市形象提升。持续开展常德优质农产品专场推介活动，合同销售金额达147.7亿元以上，常德优质农产品成功进入上海、广州、新加坡等国内外市场。编印常德工业品名录，成功举办常德工业品推介暨产销对接会，签订本地产品购销合同金额达102亿元。多措并举推介常德旅游形象，唱响桃花源品牌，桃花源、柳叶湖、穿紫河、枫林花海等频频亮相央视等主流媒体，外地团组来常游客增长近4倍。

产业扶持扎实有效。秉持“成就企业家、厚待投资者”理念，全面落实帮扶工业企业十条、降费减负等政策，为企业减负过4亿元。出台支持基金小镇发展、企业上市（挂牌）、仿制药一致性评价扶持等政策，全省第二家基金小镇柳叶湖清科基金小镇建成运营，基金规模达150.5亿元。“暖企行动”取得实效并被《湖南日报》推介。发挥重点企业微信群等服务平台作用，对企业诉求第一时间回应、第一时间交办、第一时间解决。开展涉企服务活动近100场（次），通过公交线路定制、“春风行动”系列招聘、用电企业网格化服务、企业连心卡等系列措施，解决企业反映的突出问题300多项。开展行长结对服务中小企业行动，对106家结对企业发放贷款11.8亿元。

一年来，我们大力破瓶颈、增活力，改革开放不断深入。坚持向改革要动力，向开放要活力，体制机制进一步理顺，开放格局进一步形成。

各项改革稳步实施。供给侧结构性改革、“放管服”改革持续深化。机构改革、商事制度改革“证照分离”、供销合作社改革、农村集体资产产权制度改革、新型城镇化综合改革等走在全省前列。国地税征管体制改革、国有企业“三供一业”分离移交工作有效推进。“城市双修”、海绵城市建设、社会信用体系建设等试点和改革产生较大影响。

招商引进成果丰硕。创新开展异地孵化招商，设立常德科创未来（中关村）智造中心，入驻落户高科技企业13家。举办“智汇洞庭·科创常德”系列活动，签订科技成果转移转化项目55个。签约引进亿元以上产业项目243个，对接引进长株潭绿心区转移企业20家，上海复星、青海藏格、法国施耐德、香港加多宝、正大集团等战略投资者落户常德，引进内外资总额855亿元。

开放水平显著提升。积极对接“一带一路”战略，德国汉诺威常德中心开业。推进口岸提效降费，通关时间压缩1/3，集装箱进出口环节合规成本降低100美元以上，盐关口岸集装箱吞吐量近2万标箱，增长43%。桃花源机场新增10个航点，通航城市累计达18个，旅客吞吐量达75万人次，增长71.6%，呈现跨越发展态势。完成进出口总额15亿美元，增长30%。获评全省发展开放型经济优秀市州。

要素资源保障有力。引导金融机构扩大信贷投放，新增各类贷款209亿元。强化土地供给，有力保障了重点项目建设。强化人才和科技支撑，创新实施免费报考、就近参考、小分队宣传等措施引进人才，引进博士51名、全日制硕士426名，签约到岗科技创新人才22人；常德经开区成功创建国家级科技企业孵化器，新增省级技术创新平台6家、省级科技企业孵化器9家、院士工作站2家；全年专利授权量突破2000件，增长20%以上。

一年来，我们大力强基础、提品质，城乡融合步伐加快。统筹城乡发展，提升功能品质，全市城镇化率达53%。

城镇提质亮点纷呈。成功获评全球首批国际湿地城市、中国美丽山水城市。圆满承办2018中国新型城镇化经验交流会。顺利通过全国卫生城市周期复审。海绵城市建设亮相国家庆祝改革开放四十周年大型展览。启动新一版城市总体规划（2020-2035）修编。完成棚户区改造34721户，在市城区推出一批惠民商品房，房地产市场稳健发展。永安公园市民文体广场、桃花源大桥北端棚户区改造、沅江隧道、丹溪路等重点项目有序推进。加快建设公共停车场，创新实施“路长制”，城区交通拥堵有效缓解。县（市）城区和小城镇建管加速提质，津澧新城总体规划获省政府批复。

乡村振兴全面铺开。完成自然村通水泥路建设2831公里、农村公路窄改宽748公里，实施危桥改造135座、安防工程1100公里，临澧县获批全省首批城乡客运一体化示范县。建设各类水利工程3.3万处。完成农村危房改造13529户。完成366个村电网改造。全市流转耕地面积353.9万亩，占总面积的53.8%。农村人居环境整治全域启动，美丽乡村建设不断深化，汉寿县、澧县、石门县通过全省农村环境综合整治整县推进验收。推动以文明节约为主题的移风易俗工作，乡村社会文明程度不断提高。

交通建设加力推进。完成交通投资80亿元。黔张常铁路进入全线铺轨阶段，常益长高铁征地拆迁、节点性工程设计顺利推进，常德铁路枢纽站完成初步设计方案，安慈、官新高速公路等项目加快推进。沅水浦市至常德航道整治完成主体工程，澧县至安乡至茅草街航道全线贯通。新改建干线公路138公里，沅澧快速干线2号大道临澧段、6号大道常德经开区段等公路建成通车，319和207国道市城区改线工程南段、常德沅水四桥、安乡长岭大桥、津市澧水二桥、石门杨岭岗澧水大桥和桃源陬市、津市窑坡渡码头完成主体工程。公路治超保持“六连冠”。

一年来，我们大力抓脱贫、惠民生，人民生活持续改善。以打好精准脱贫攻坚战为统揽，全力保障和改善民生。全市财政用于民生支出达395亿元，占一般公共预算支出的75%。城乡居民人均可支配收入分别达31063元、15093元，增长8.1%、9%。

脱贫攻坚精准有效。聚力打好产业发展、住房安全、教育助学、健康扶贫、就业创业、基础建设等“六场硬仗”，强化帮扶责任等保障措施，整合各类财政资金12.08亿元，累计发放扶贫小额信贷16.3亿元，完成易地扶贫搬迁8704人，新增贫困劳动力转移就业7004人。深入开展扶贫领域作风问题专项治理，查处了一批典型案件。全年脱贫5.4万人、128个村退出贫困村序列。石门县成功脱贫摘帽，成为全省脱贫样板。

教育质量持续提升。改造农村薄弱学校166所，建成标准化学校36所，新建普惠性幼儿园96所。国家义务教育基本均衡县创建全市达标，超大班额全面消除。湖南师范大学附属德山学校建成招生。高考成绩再创新高。高等教育、职业教育、特殊教育稳健发展。桃源一中以德治校经验被央视等媒体推介。

社会保障不断加强。新增城镇就业5.87万人、失业人员再就业3.1万人、创业带动城乡就业4.5万人。新增创业担保贷款2.5亿元。五项社会保险新增扩面15.8万人次。企业职工和城乡居民基本养老金、城乡低保等待遇水平持续提高。

各项事业全面进步。综合医改试点持续推进，全市二级以上医疗机构开通预约诊疗渠道。计划生育失独和伤残家庭补助标准大幅提高。公共文化服务体系建设得到加强。2018常德柳叶湖国际马拉松赛成功举办，我市运动健儿在第十八届亚运会、第十三届省运会上取得优异成绩。统计、外事侨务、民族宗教、对台、侨联、住房公积金、贸促、残联、老龄、方志、档案等工作取得进步，工会、共青团、妇联作用充分发挥。关心下一代工作创新开展。国防动员、国防教育、军事设施保护、人防建设、民兵预备役和双拥工作持续加强，民兵集中轮训试点经验全省推广。

一年来，我们大力治污染、重保护，生态建设强力推进。切实打好污染防治攻坚战，坚持铁腕整治、一抓到底，较好完成中央生态环保督察“回头看”和省生态环保督察问题整改、“2018夏季攻势”等工作年度任务。

污染防治扎实开展。切实抓好污染治理，实施治理项目498个。深入开展洞庭湖生态环境专项整治，清理砍伐西洞庭湖自然保护区欧美黑杨2.1万亩，清理砂石码头206处，拆除网围99.6千米、矮围164千米、网箱102.8万平方米。整治黑臭水体29条，珊珀湖水质持续改善。烟花鞭炮禁燃、秸秆禁烧、农业面源污染治理、餐饮油烟整治、鼎城石板滩石煤矿山治理等扎实推进。

督察整改取得实效。中央生态环保督察“回头看”交办群众信访举报271件，已办结215件；省生态环保督察交办群众信访举报503件，已办结489件，办结率居全省前列。238家黏土砖厂全部关闭，全面完成44家石膏矿山整治，年内所有煤矿可关闭退出。关闭“散、乱、污”企业190家、整改101家，洞庭湖区造纸企业引导退出工作加快推进，省级以上工业园区污水处理设施全部投入运行。

生态环境总体改善。全面推行河（湖）长制，对主要江河湖库开展规范性管护，23个省控及以上考核断面水质Ⅲ类以上达95.7%，县级及以上9个饮用水水源地水质全部达标。完成大气污染物排放源清单编制，市城区环境空气质量优良天数达290天，增加15天。土壤污染防治先行区建设成效明显。完成人工造林24万亩，义务植树1134万多株。石门县入选全国森林康养试点县，安乡县成功创建全国渔业健康养殖示范县，澧县涔槐国家湿地公园试点通过国家验收。

一年来，我们大力防风险、保安全，社会大局和谐稳定。坚持打好防范化解重大风险攻坚战，各类风险隐患得到有效防控。

债务化解稳步推进。强化制度建设，统筹推进债务清理、项目压减、存量消化等工作。全面禁止违规举债，按照“停、缓、调、撤”要求，严控项目规模数量，压减投资392亿元。盘活国有资产和要素资源，加快平台公司整合转型，债务风险安全可控。

安全生产形势向好。全面启动国家安全发展示范城市创建，深入开展安全生产打非治违百日行动，对重大事故隐患整改和462个典型案例公开曝光，发布安全生产一号追逃令，安全生产责任进一步压实。开展国家农产品质量安全市、国家食品安全示范城市创建，扎实推进农产品、食品药品安全体系和工程建设，连续16年实现农产品质量安全零事故，连续17年未发生重大食品药品安全事故。全力打好非洲猪瘟防控战，桃源县疫区解除封锁。

社会治理能力提升。扎实推进平安常德建设，综治民调排名全省前列。全力推进无上访村（社区）建设，矛盾纠纷有效化解。深入开展扫黑除恶专项斗争，治安形势稳定向好。全面推行雪亮工程，武陵区雪亮工程建设获评全国十大创新案例奖。

一年来，我们大力改作风、优环境，政府建设全面加强

政务服务明显改善。大力优化经济发展环境，重拳出击整治行政执法、项目建设等环境问题，对窗口服务暗访曝光，55家单位109名当事人被追责问责。大力推进“互联网+政务服务”，实施流程再造和综合服务窗口改革，取消257项证明（盖章）类材料，577项办事事项实现“最多跑一次”，15项政务服务事项下沉到乡村，武陵区被列为全国基层政务公开标准化规范化试点单位。企业开办时间压缩至5个工作日以内，各类市场主体达28万家，增长24.5%。

依法行政深入推进。自觉接受市人大及其常委会的法律监督、工作监督以及市政协的民主监督，定期向市人大报告工作，向市政协通报情况，主动向市人大常委会提请审议政府重大项目情况，执行市人大及其常委会的决议决定，积极研究处理各类审议意见，全年办理人大代表建议260件、政协提案336件。认真吸纳各民主党派市委、无党派人士和工商联调研成果。严格依法科学民主决策，完善政府投资项目管理、市属企业国有资产监督管理等制度机制。“七五”普法中期督导顺利验收。

政风作风切实改进。严守政治纪律和政治规矩，深入推进“两学一做”学习教育常态化制度化。严格遵守改进作风各项纪律规定，进一步规范“三公”经费支出，切实强化审计监督，政府系统党风廉政建设进一步加强。大力推进主动公开，加大行政执法典型案例、热点难点问题解决等事项公开力度，以公开促监督、以公开促公正、以公开促公信。大力践行“一线工作法”，坚持深入一线调查研究、深入一线攻坚克难、深入一线督促落实。大力倡导立说立行，以认真较真的态度，加大交办、催办、督办力度，确保重大决策、重点工作、重要事项压茬推进。政府系统清正清廉、担当作为的氛围进一步形成。

各位代表！过去的一年，我们在诸多压力交织叠加、多重困难挑战并存的形势下，保持定力强担当，着眼长远谋发展，立足当前抓推进，创新思路解难题，取得了来之不易的成绩。这是习近平新时代中国特色社会主义思想指引的结果，是省委省政府和市委正确领导、科学决策的结果，是市人大、市政协大力监督、鼎力支持的结果，是全市人民共同努力、携手奋斗的结果。在此，我代表市人民政府，向积极投身常德经济社会发展的广大干部群众、驻常部队和武警官兵，向中央、省驻常单位，向各位人大代表和政协委员，向各民主党派、人民团体、工商联和社会各界人士，向所有关心支持常德发展的同志们、朋友们，表示衷心的感谢！

在看到成绩的同时，我们更应该清醒地看到，常德发展还面临不平衡不充分的问题，政府工作还存在一些需要加强和改进的地方。主要表现在：产业结构性矛盾仍然存在，重大产业项目不多，园区实力不强，科技创新对经济发展的带动还不够；县域经济、民营经济、开放型经济仍然是发展的短板，金融服务实体经济的能力还有待增强；三大攻坚战任务仍然艰巨，民生领域还存在一些问题和不足；政府自身建设仍然有待加强，一些干部担当精神、服务意识不足，创新精神和落实能力有所欠缺，经济发展环境需要进一步改善，腐败问题和不正之风还不同程度存在。对此，我们将高度重视，采取有效措施，切实加以解决。

二、2019年工作安排

2019年，是新中国成立70周年，是全面建成小康社会关键之年。当前，我国经济运行稳中有变、变中有忧，外部环境复杂严峻，经济面临下行压力，区域竞争日趋激烈。我们要辩证看待国际环境和国内条件的变化，充分认识到我国发展仍处于并将长期处于重要战略机遇期，经济长期向好的态势不会改变，充分认识到“一带一路”、长江经济带、中部地区崛起和洞庭湖生态经济区等国家战略深入推进的大好机遇，坚定信心，把握主动，积极稳妥应对各种挑战，牢牢把握重要战略机遇期新内涵，推动常德经济社会发展迈上新台阶。

做好政府工作，必须坚定决战决胜全面小康目标不动摇。围绕人民向往，全力抓重点、补短板、强弱项，确保如期建成“五位一体”全面进步的小康、城乡区域共同发展的小康、惠及全体人民的小康。必须践行高质量发展的根本要求不动摇。深入贯彻新发展理念，坚持质量第一、效益优先，确保经济运行在合理区间，实现更高质量的发展。必须把握稳中求进的工作总基调不动摇。保持定力，把握好节奏和力度，进一步稳就业、稳金融、稳外贸、稳外资、稳投资、稳预期，提振发展信心。必须坚持改革创新的思维路径不动摇。传承改革创新的时代精神，以问题为导向推进改革，大力破除体制机制障碍，有效化解发展中的困难和问题。必须推进开放强市产业立市战略不动摇。准确把握常德的市情特征，坚定不移在扩大开放中谋合作、在壮大产业中促跨越，加快发展开放型经济，积极构建现代产业体系，努力建设省域副中心城市和现代化区域中心城市。

根据新的形势和任务，政府工作的总体要求是：以习近平新时代中国特色社会主义思想为指导，全面贯彻落实党的十九大、十九届二中、三中全会精神和中央、省委、市委经济工作会议精神，统筹推进“五位一体”总体布局，协调推进“四个全面”战略布局，坚持稳中求进工作总基调，坚持新发展理念，坚持推动高质量发展，坚持以供给侧结构性改革为主线，坚持深化市场化改革、扩大高水平开放，深入实施创新引领开放崛起战略，大力推进开放强市产业立市，继续打好三大攻坚战，统筹推进稳增长、促改革、调结构、惠民生、防风险工作，确保经济持续健康发展和社会大局稳定。

经济社会发展的主要预期目标是：地区生产总值增长8%，规模以上工业增加值增长7%以上，固定资产投资增长11%，一般公共预算收入增长6.5%以上，城乡居民人均可支配收入增长8.5%。全社会研发经费支出占地区生产总值1.9%，产业投资占固定资产投资53%，地方财政收入中税收占比71%。

实现上述目标任务，必须做好以下九个方面的工作。

一、聚焦产业行动，加快提升发展质量。持之以恒推进产业立市三年行动，着力壮大实体经济，提高经济发展质效。

促进产业升级。扎实推进产业项目建设，突出抓好列入全省“五个100”的项目，新开工亿元以上工业项目100个以上，力争烟厂易地技改、中联重科起重机械产业基地、中国中药产业园、金康光电等一批项目竣工投产。提质改造传统产业，推动烟草、纺织、酿酒、建材等产业“老树发新枝”。积极培育新兴产业，加快发展装备制造、健康食品、生物医药、新材料、新能源等优势产业链。大力发展先进制造业，支持企业加强技术改造和设备更新，培育一批智能制造示范企业、示范车间和绿色园区、绿色工厂。

做强产业园区。推动园区走特色化、集约化、人性化发展道路。支持特色园区发展，全面落实优化园区产业布局指导意见，出台专门支持政策，加快建设常德经开区装备制造与军民融合产业园、常德高新区工程机械配套产业园、临澧新材料产业园、津市生物医药与健康食品产业园、桃源电子信息产业园、武陵智慧产业园等特色园、园中园，引导园区培育一批“镇园之宝”。继续加强园区水电路气等基础设施建设，抓好园区商业、餐饮、娱乐、出行等生活服务设施配套，力争园区完成基础建设投入100亿元以上，建设标准化厂房100万平方米以上。

狠抓品牌培育。分行业常态化开展“常德品牌·中国品质”行动，提高常德品牌影响力，让世界爱上常德造。引导企业增强品牌意识，加大品牌推介力度，参与制订修订国家、行业标准体系，积极争创中国质量奖、省长质量奖，培育更多“常德工匠”和“百年老店”。实施农产品“三品”工程，改良品种，提升品质，增强品牌附加值和影响力。加强香米、茶油、茶叶、甲鱼、米粉等区域公用品牌培育。支持桃花源、城头山创建国家5A级景区，柳叶湖创建国家级旅游度假区。

推动产业融合。促进先进制造业与现代服务业深度融合、消费与投资一起拉动经济增长。统筹抓好文旅康养产业发展，开发“旅游+”产品，以旅行商为纽带，实施精准营销，办好旅游节会活动，培育新的消费热点，确保卡乐星球、大唐司马、清水湖野生动物园等一批项目顺利建成，全年旅游总收入增长15%以上。大力引进金融机构，运营好柳叶湖清科基金小镇，推动产业基金设立相关子基金，把更多的金融“活水”引到实体经济发展上来，力争新增贷款200亿元以上。加快企业上市步伐，力争新增1家企业在主板上市。大力发展现代物流、电子商务、检验检测等生产性服务业。社会消费品零售总额增长10%以上。

支持民营经济。全面贯彻落实习近平总书记在民营企业座谈会上的重要讲话精神，对上级减税降费等政策，坚决不折不扣落实到位；对包括民营企业在内的各类所有制企业参与市场竞争，坚决做到一视同仁；对民营企业和民营企业家的合法权益，坚决保障到位，让民营企业扎下根来，安心谋发展。出台支持民营经济发展的政策措施，减少对资源的直接配置，全面实施市场准入负面清单制度，支持民营企业在政府采购和政府投资项目建设中发挥重要作用。降低融资担保门槛和费率，建立民营企业创业风险救助基金，让企业家放下包袱，大胆经营。持续深入开展“暖企行动”，完善领导干部联系重点企业制度，实施精准服务，全力为企业排忧解难。

各位代表，民营经济是推动市场经济发展的重要力量，是常德高质量发展的重要主体，各级政府及其部门要坚定不移地支持民营经济发展，让市场自主调节，让企业大显身手，保障和激励广大企业家在市场经济的大潮中抢抓机遇、勇立潮头！

二、打赢攻坚战役，努力跨越发展关口

有效防范化解重大风险。有效遏制债务增量，严禁违法违规融资担保，严禁变相举债，严格管控新增项目融资行为。全力化解债务存量。加强投融资平台公司管理，强化资产负债率约束，完成平台公司转型整合。规范政府投资项目管理，严格按政策规定推进PPP和政府购买服务项目。用好用活地方政府债券，保障正常融资需求。打击和处置非法集资，加强金融风险防范宣传，确保地方金融稳定。

精准推进扶贫脱贫。把提高脱贫质量、巩固脱贫成效摆在突出位置，实现贫困村全部出列、除兜底保障外的贫困人口全面脱贫。因地制宜推进产业扶贫，注重依托优势资源，遵循市场规律，实现村村有扶贫产业、户户有增收项目。加强贫困人口技能培训，加大教育助学力度，阻断贫困代际传递。继续抓好交通、电力、饮水、通讯、医疗、住房安全等普惠性工程，推动基础设施和公共服务上台阶。引导贫困群众依靠双手实现脱贫致富，激发脱贫内生动力。

切实加强污染防治。巩固环保整治成果，坚决抓好中央生态环保督察“回头看”和省生态环保督察问题整改工作，切实打好蓝天、碧水、净土保卫战。加强大气污染联防联控，强力推进污染源综合治理，确保市城区环境空气质量优良率达到78%以上。统筹推进“一湖两水”生态环境保护和综合治理，深入实施河（湖）长制，重点实施农业面源污染、城乡生活污染、工业污染、黑臭水体等领域治理，确保省控及以上地表水考核断面水质优良率达到88.3%。开展耕地修复治理、矿山生态修复，推进土壤污染防治先行区建设，抓好第三次国土调查工作。积极创建生态文明建设示范市，统筹山水林田湖草系统治理，加强湿地保护修复，推进西洞庭湖自然保护区立法工作。实行最严格的生态保护制度，推动形成绿色发展方式和生活方式。

三、致力扩大开放，不断拓展发展空间。坚定不移解放思想，走开放强市之路，以高水平开放推动高质量发展。

突出开放“大引进”。进一步强化招商引资主体责任，加强招商项目策划和研究，抓好招商引资管理和服务。持续开展节会招商，积极参加“对接粤港澳大湾区”等招商活动，创新德商恳谈会活动形式，打造德商回乡产业园，引导德商总部回归、产业回归、资本回归和人才回归。鼓励开展结对招商，与发达地区园区、商会、企业开展深度合作，承接产业转移。大力开展异地孵化招商，发挥常德科创未来（中关村）智造中心带动作用，支持区县（市）、园区在一线城市设立招商孵化平台。积极开展驻点招商，组建驻外招商中心，打造专业化招商队伍。探索开展委托招商，提高市场化招商水平。全年实际利用外资增长10%以上，引进亿元以上产业项目200个、5亿元以上产业项目60个，力争在引进“500强”等战略投资者上实现突破。

搞活开放“大市场”。积极推进德企德品出海行动，依托龙头企业，对接“一带一路”，在俄罗斯、东南亚等国家和地区举办常德品牌推介活动，推动优势行业、优秀企业和优质产品走出去。优化进口贸易方式，加快发展跨境电商等新业态，促进本土企业转型升级。力争进出口总额增长20%以上。

建设开放“大通道”。确保黔张常铁路竣工通车，加快常益长高铁、常德铁路枢纽站和安慈、官新高速公路建设，抓紧襄常高铁、益常高速公路扩容工程前期工作。力争开通常长城际公交，大幅压缩融入长株潭的时空距离。新改建干线公路100公里以上，确保沅澧快速干线4号大道桃源段、5号大道西洞庭段等公路建成通车。提质改造省际边界口子镇主干道。推进沅澧水航道建设，依托常德港务集团整合全市码头资源，加快复兴水运优势。支持石门县、澧县等通用机场建设。开通新的机场航班航线，加密热点城市航线班次，力争旅客吞吐量突破100万人次。

提升开放“大平台”。加强口岸建设，保持“五定班轮”常态化运营，推进常德港集装箱码头建设，确保桃花源机场临时航空口岸开放，继续申建常德保税物流中心（B型）和铁路口岸。打造汉寿县、石门县农产品出口示范区，助推泰中东盟建材产业经贸园建设。支持外贸平台企业开展一站式综合服务，大力压缩通关时间、简化单证和降低成本。

各位代表，思想是行动的先导，开放是时代的强音。站在改革开放40周年的新起点上，我们一定要以更开阔的视野、更包容的姿态、更坚定的步伐，全方位融入开放大格局，全力开创常德发展新局面！

四、实施乡村振兴，着力夯实发展基石

发展现代农业。全面落实国家粮食安全战略，大力实施“优质粮食工程”，积极推广“稻田+”、林下经济等生态种养模式，着力增加优质绿色农产品供给。积极培育新型经营主体，新增家庭农场1000个以上、农民合作社200家以上。大力推进现代农业园区、农业特色产业小镇建设，加强农业科技推广应用，引导产业向产区、园区集中，努力创建一批国家级、省级特色农产品优势区，力争农产品加工业销售收入突破1450亿元。加快推进“互联网+现代农业”，拓宽农产品线上流通渠道。促进农业与文化旅游、生态休闲深度融合。

建设美丽乡村。扎实推进农村人居环境整治三年行动，重点做好垃圾污水处理、厕所革命、村容村貌提升。加快推进乡村振兴“4+9”示范片建设、美丽乡村示范村创建和县乡全域推进试点，完成120个村的农村环境综合整治任务。实施乡镇人居环境整治试点，加强以有效保洁、定点停车、店内经营、违建拆除等为重点的镇容镇貌管理。依托环境整治，培育和引进生态经济主体，打造319国道（常德）百里生态经济走廊。开展城乡建设用地增减挂钩暨农村“空心房”整治，依托自然村落、集中安置点合理引导农民集中居住。培育文明乡风、良好家风、淳朴民风。

改善基础条件。加强农田水利基础设施建设，推进农村土地整治和高标准农田建设，实施农村饮水安全巩固提升工程，抓好澧县安乡西水东调工作。高质量推进“四好农村路”建设，实施农村公路窄改宽500公里，全面完成自然村通水泥路建设。加快新一轮农村电网改造升级，加强乡村通信基站和光缆线路建设。

夯实基层基础。大力发展壮大村级集体经济，增加村级公共服务自给能力。加大基层组织运转经费保障力度，保障好基层干部待遇。充分发挥自治章程、村规民约在乡村治理中的作用，建立健全现代乡村社会治理体制。

五、推动城镇提质，持续完善发展平台

提升城市功能。强化规划引领，坚持多规合一，继续抓好新一版城市总体规划修编，加强柳叶湖空间规划管理。全面推进“城市双修”，巩固海绵城市建设成果。加快城东片区提质改造、桃花源大桥北端棚户区改造、高铁新城、阳明湖板块、新河水系整治等重点项目建设，推进沅江隧道、太阳大道、丹溪路等骨干路网建设。大力推进“公交都市”创建，支持市域内现有客运班线升级为城际公交。加快市城区停车设施规划建设，改善城市交通环境。规范房地产市场秩序，促进房地产市场平稳健康发展。

加强精细管理。推动城市管理拓展延伸，提高科学化、精细化、智能化管理水平。严格落实《常德市城乡生活垃圾管理条例》，加强垃圾分类处置。完善出租车改革后续管理长效机制。提高市政设施、园林绿化管养水平。持续抓好控违拆违。加快拓展“我的常德”城市公共服务平台功能，积极创建国家新型智慧城市示范城市。巩固城市创建成果，提高市民素质和社会文明程度。

完善城镇体系。统筹城市和城乡规划布局，推进县域品质提升，增强县域辐射带动作用，促进县域产城融合发展。加快推进城镇体系规划编制管理，培育发展特色小镇和品牌村落。积极探索扩权强镇，加快农业转移人口市民化进程。

六、深化改革创新，有效激发发展活力

抓好重点改革。扎实推进供给侧结构性改革，持续实施“放管服”改革，加快推进农村综合改革。统筹抓好机构改革和事业单位分类改革，确保平稳顺利推进。深化国有企业改革，加快完善现代企业制度和市场化经营机制。支持园区体制改革试点，让园区拥有更多的管理审批、选人用人、薪酬改革自主权，支持县级探索开展“园镇合一”改革。探索实施江北城区市区两级建设管理体制调整，实行“三统四分”，增强区级积极性和主动性。

推进科技创新。积极引进建设一批国家、省级重点实验室、工程（技术）研究中心和科技企业孵化器，新增院士工作站2家以上，推动建设院士小镇。新增高新技术企业40家，高新技术产业产值达到1600亿元。分行业区域举办“智汇洞庭·科创常德”系列活动，引导企业对接科研院所和知名高校，全年对接不少于30家，促成一批重大科技成果在常德落地转化。落实科技创新政策，发挥财政资金的引导作用，鼓励全社会加大科技研发投入，强化知识产权创造、保护和运用。大力发展军民融合产业。积极推进“双创”平台建设。

加强人才培育。开辟人才引进“绿色通道”，积极引进一批产业精英人才、高技能人才和职业经理人，大力弘扬劳模精神、工匠精神和企业家精神。建立更为灵活的人才管理机制，保护尊重人才，重视培养人才，畅通流动通道，优化服务保障，让各类人才的创造活力竞相迸发、聪明才智充分涌流。

七、提升县域经济，积极增强发展实力

强化分类指导。加强统筹指导，促进县域特色发展、错位发展、加快发展。推动市城区“一江两岸”协调发展。推进津澧融城，加快常桃融合，打造市域副中心。深入实施“两西”发展战略，支持建设常德卫星城镇。推动县域依托区位、产业优势，找准发展着力点，形成各展所长、竞相发展的格局。

加大支持力度。充分发挥基础设施对县域发展的支撑作用，进一步改善交通、水利、能源等领域基础设施，夯实县域发展基础。充分发挥生产要素对县域发展的保障作用，支持金融机构向县域下沉，在土地、人才引进等方面对县级予以倾斜，促进人才、资金、技术向县域流动。充分发挥产业项目对县域发展的带动作用，在项目布局、引进战略投资者等方面给予更多支持，设立特色产业发展基金，支持和引导县域特色优势产业发展。

激发内生动力。深入推进扩权强县，进一步理顺市、县事权与支出责任，增强县域发展主体主责意识。着力增强乡镇经济基础，鼓励引导乡镇发展乡村旅游、生态农业、健康食品等绿色产业，对成效明显的乡镇在特色产业基金中予以支持。探索实施分类绩效考核，加强激励约束，促进县域发展对标赶超、争先进位，激励县（市）进入全省县域经济十强、二十强。

八、增进民生福祉，全面共享发展成果

坚持教育优先发展。扩大教育资源供给，严格落实新建小区配套建设学校的各项规定，加强市县城区中小学校学位和公办幼儿园建设，支持普惠性民办幼儿园发展，北部新城实验学校、武陵区第一小学等建成招生。完成大班额年度化解任务。坚持立德树人，发展素质教育。加强教师队伍建设，切实保障教师待遇，均衡配置教师资源，树立良好师德师风。促进教育协调发展，支持发展高等教育、民办教育、特殊教育和老年教育，引导职业教育服务地方发展。规范校外培训和托管辅导机构管理。大力实施农村学校建设三年行动计划，让农村留得住青年教师，让学生吃得上热饭热菜，让学生有场地锻炼健康体魄。

加快建设健康常德。深化医药卫生体制改革，巩固综合医改成果，大力引进名医，积极做好湘雅常德医院、市妇幼保健院及县级重点医院创建升级工作。加强县级医院综合能力建设和乡镇卫生院、村卫生室规范化建设，大力培养全科医生。加强医德医风建设。扎实做好急性传染病和慢性病防治工作。传承发展中医药事业。深入开展爱国卫生运动。积极推进医养结合，大力发展养老事业。加强优生优育和健康服务管理，降低出生缺陷发生率。

加强就业和社会保障。把稳就业摆在突出位置，重点解决好高校毕业生、农民工、退役军人等群体就业，新增城镇就业5.6万人、农村劳动力转移就业3.3万人。统筹推进社会保险制度改革，加强各类社会保险征缴扩面工作。完善社会救助保障体系，深入推进全国居家和社区养老服务改革试点，稳步提高城乡低保标准和救助水平。关爱失独家庭、农村留守人员，重视老龄事业发展，加强残疾人康复服务，发展残疾人事业。

大力发展文体事业。坚持文化惠民，推进市体育中心提质改造及社区体育中心建设，加快村级综合文化服务中心建设。丰富群众性文体活动，办好常德柳叶湖国际马拉松赛等大型赛事。加强文艺精品创作。推动文物保护与利用，加强非物质文化遗产挖掘与传承，积极推进鼓书文化省级生态保护（试验）区创建。

统筹发展其他事业。做好统计、外事侨务、民族宗教、对台、住房公积金、方志、档案、保密、慈善等工作。支持工会、共青团、妇联等群团组织工作。深化双拥共建，提高退役军人服务管理水平，抓好国防教育、军事设施保护、人民防空、民兵预备役和兵役征集工作。

在全面完成省重点民生实事的基础上，突出抓好十项重点民生实事。①实施农村学校建设三年行动，新建教师周转房3307套，新建和改扩建学生食堂70619平方米，新建塑胶运动场105个，年内完成1/3以上；②加快发展普惠性幼儿园，增加园位7000个；③加快实施农村危桥改造工程，改造农村危桥100座，力争完成130座；④推进农村交通事故易发多发点改造整治，完成隐患路段整改310公里、事故多发点整治38处；⑤强化施工管理，有效解决噪音扰民问题；⑥大力规范出租车文明营运，整治车容车貌、拼客宰客、择客拒载、野蛮行车、态度恶劣等问题；⑦加强小作坊、小餐饮、小摊贩食品安全监管，提升食品安全水平；⑧加强住宅小区物业管理，改善小区居住环境；⑨抓好生活垃圾与餐厨废弃物处置，建立规范化长效化机制；⑩开展城镇和农村村庄规划编制，完成城镇规划编制50个、村庄规划编制880个。

九、优化社会治理，切实强化发展保障

创新治理体系。推进完美社区、智慧常德、智慧党建等平台深度融合，完善综合服务管理，健全基层群众自治制度，推动社会治理重心向基层下移。积极创建社会信用体系建设示范城市，加强诚信评级评价和失信惩戒。完善社会组织管理。健全立体化、信息化社会治安防控体系，推进“一村一辅警”“城市快警”建设。深化平安常德建设，打造共建共治共享的社会治理格局。

保障公共安全。持续推进国家安全发展示范城市、国家农产品质量安全市和国家食品安全示范城市创建。严格落实安全生产责任制，加大专项整治、风险分级管控、隐患排查和公开曝光力度，坚决遏制较大及以上生产安全事故发生。提升食品药品和农产品质量安全监管能力，深化重点领域、重点环节、重点品种专项整治。深入推进扫黑除恶专项斗争，依法严厉打击涉毒、涉枪、多发性侵财、涉众性经济犯罪等违法犯罪行为。积极抓好防汛抗旱，实现“五个确保”目标。做好防震减灾、森林防火、城乡消防、动植物疫病防控等工作，提高公共应急管理水平。

维护社会稳定。完善社会矛盾排查预警、矛盾纠纷多元化解机制。加强信访法治建设，加大领导干部接访下访力度，依法及时解决群众合理诉求。深化无上访村（社区）建设，构建市县乡村四级联调体系，切实把矛盾纠纷化解在基层、处置在萌芽状态。加强公共法律服务体系建设，加大法律援助力度，保障困难弱势群体合法权益。

三、全面加强政府自身建设

民之所望，政之所向。各级政府要始终坚持以人民为中心，牢记职责使命，强化责任担当，增强履职能力，提升服务水平。

牢记宗旨，忠诚履职。各级政府及工作人员务必坚持对党绝对忠诚的政治品质，牢固树立“四个意识”，坚决维护习近平总书记党中央的核心、全党的核心地位，坚决维护党中央权威和集中统一领导。坚持把中央大政方针和省委、市委决策部署落实到行动、体现在工作、彰显于业绩。始终牢记政府前面的“人民”二字，恪守为民之责，善谋富民之策，多办利民之事，以政府的决心换取人民的舒心，以干部的真情换取群众的真心。

坚守法治，依法履职。树牢法治理念。严格遵守宪法法律，坚持权由法定、权依法使，自觉运用法治思维和法治方式推进工作。规范行政行为。健全科学民主依法决策机制和社会稳定风险评估机制，深化行政执法体制改革，严格规范公正文明执法。自觉接受监督。自觉接受市人大及其常委会的法律监督和工作监督，执行市人大及其常委会的决议决定，认真研究处理各项审议意见，主动接受市政协的民主监督，认真办理人大代表建议和政协提案，虚心听取和采纳各民主党派、工商联、无党派人士的意见建议。深化政务公开。阳光是最好的防腐剂，公开是最好的监督。加大政策事项、民生改善、行政执法、工作落实等方面的公开力度，在政府工作中树立不发现问题就是失职，发现问题不研究解决、不通报曝光就是渎职的工作导向。

优化服务，高效履职。持续推进简政放权。进一步精简行政审批，最大限度下放行政审批权限，减少不必要的行政审批中介服务事项，为市场添活力，为发展增动能。加快推进“互联网+政务服务”。推动流程再造，压缩办理时限，扩大网上服务事项，拓展网上服务方式，加快实现“一网通办”“只进一扇门”“最多跑一次”，让企业和群众到政府办事像“网购”一样方便。强力推进发展环境整治。对标先进地区和群众需求，下猛药、去沉疴、挥重拳，建立完善工作机制，大力推进重点整治，对影响发展环境的突出问题采取果断措施，坚决查处违规违法行为，全力打造公平开放透明市场规则和法治化营商环境。

敢于作为，担当履职。任务越艰巨，担当越可贵。面对矛盾困难，各级政府及工作人员必须知难而进、迎难而上、破难而行。坚持认真较真。以认真的态度奋发干事，以较真的态度动真碰硬，说了的坚决做到，定了的坚决执行。强化政府工作问效问责，严防责任口头化、书面化、图章化。坚持严管厚爱、激励约束并重，保护好干部的积极性、主动性、创造性。坚持务实扎实。大力践行“一线工作法”，倡导说干就干、马上就干、干就干好的工作作风，着力解决表态多调门高、行动少落实差等问题，确保事事有着落、件件有回音。积极应对重大挑战、抵御重大风险、解决重大矛盾，争做“主攻手”、不当“二传手”。坚持创新创优。坚决破除思维定势和路径依赖，注重用创新的思维寻求突破、用改革的办法破解难题，促进政府工作创新求变、变中求进。

清正廉洁，干净履职。把全面从严治党贯穿于政府建设始终，认真落实党风廉政建设主体责任和“一岗双责”，深化廉政风险防控，抓好谈心谈话工作。加强审计监督，加大公共资金、国有资产、国有资源监管力度，规范公共资源交易，把制度的笼子扎得更紧、更密、更牢。自觉遵守中央八项规定及实施细则，驰而不息整治“四风”，力戒形式主义、官僚主义及其隐形变异，大力倡俭治奢、防微杜渐，切实树立清新政风。

各位代表！征程万里风正劲，重任千钧再扬鞭。让我们更加紧密地团结在以习近平同志为核心的党中央周围，在省委省政府和市委的坚强领导下，奋力推进开放强市产业立市，以优异成绩迎接新中国成立70周年，为谱写常德发展的精彩篇章而努力奋斗！
